# Comparison of pelvic floor dysfunction 6 years after uncomplicated vaginal versus elective cesarean deliveries: a cross-sectional study

**DOI:** 10.1038/s41598-020-78625-3

**Published:** 2020-12-09

**Authors:** David Baud, Joanna Sichitiu, Valeria Lombardi, Maud De Rham, Sylvain Meyer, Yvan Vial, Chahin Achtari

**Affiliations:** Materno-Fetal and Obstetric Research Unit, Woman-Mother-Child Department, University Hospital of Lausanne, CHUV, 1011 Lausanne, Switzerland

**Keywords:** Risk factors, Signs and symptoms, Urology

## Abstract

Clinicians and patients have traditionally believed that elective cesarean section may protect against certain previously ineluctable consequences of labor, including a plethora of urinary, anorectal and sexual dysfunctions. We aimed to evaluate fecal, urinary and sexual symptoms 6 years postpartum, comparing uncomplicated vaginal delivery and elective cesarean delivery, and to assess their impact on quality of life. We conducted a cross-sectional study to compare perineal functional symptomatology between women having singleton elective cesarean deliveries (eCS) and singleton uncomplicated vaginal deliveries (uVD). Women who delivered 6 years before this study were chosen randomly from our hospital database. This database includes demographic, labor, and delivery information, as well as data regarding maternal and neonatal outcomes, all of which is collected at the time of delivery by the obstetrician. Four validated self-administrated questionnaires were sent by post to the participants: the short forms of the Urogenital Distress Inventory, Incontinence Impact Questionnaire, Wexner fecal incontinence scale, and Female Sexual Function Index. Current socio-demographic details, physical characteristics, obstetrical history and mode of delivery at subsequent births were also registered using a self-reported questionnaire. A total of 309 women with uVD and 208 with eCS returned postal questionnaires. The response rate was 49%. Socio-demographic characteristics and fecal incontinence were similar between groups. After eCS, women reported significantly less urgency urinary incontinence (adjusted Relative Risk 0.55; 95% confidence interval 0.34–0.88) and stress incontinence (adjusted Relative Risk 0.53; 95% confidence interval 0.35–0.80) than after uVD. No difference in total Incontinence Impact Questionnaire score was found between both modes of delivery. Lower abdominal or genital pain (adjusted Relative Risk 1.58; 95% confidence interval 1.01–2.49) and pain related to sexual activity (adjusted Relative Risk 2.50; 95% confidence interval 1.19–5.26) were significantly more frequent after eCS than uVD. Six years postpartum, uVD is associated with urinary incontinence, while eCS is associated with sexual and urination pain.

## Introduction

Pelvic floor dysfunction is a devastating symptom with considerable negative impacts on the psychological and physical health of the patient. Some studies^1,2^ suggest that women who experience vaginal delivery have a higher risk of developing pelvic floor dysfunction than women who undergo cesarean section, while others failed to demonstrate any benefit with cesarean section. The latter promote vaginal delivery and claim that pregnancy itself is one of the major risk factors for pelvic dysfunction, at least for stress urinary incontinence^3^.

Vaginal delivery is assumed to cause pelvic floor muscle and nerve injuries, with short- and long-term damage to pelvic organs, thus inducing urinary, anorectal or sexual dysfunction, while an elective cesarean section (eCS) without labor is thought to protect against pelvic floor dysfunction. eCS is therefore often requested by patients thus potentially increasing the frequency of this procedure without proven benefit^1–4^. There is conflicting evidence as to whether or not an elective cesarean section confers protection against pelvic dysfunction. Several studies compared both modes of delivery and failed to achieve clear conclusions, either because of short follow-up period (< 1 year)^5,6^, and mixing elective and emergency cesarean sections^5–7^ or uncomplicated and instrumental vaginal deliveries^6,7^. Other studies were limited by small patient cohort^5^, or failed to analyze all aspects of pelvic floor function^5–7^. Given the latency between delivery and pelvic floor dysfunction, the association between childbirth and pelvic floor dysfunction is difficult to establish^8^.

We aimed to evaluate fecal, urinary and sexual symptoms 6 years postpartum, comparing uncomplicated vaginal delivery and elective cesarean delivery, and to assess their impact on quality of life. Validated questionnaires grading fecal, urinary and sexual function were used to evaluate the long-term impact of the mode of delivery.

## Methods

This cross-sectional study was designed using our obstetrical database at the Maternity Hospital of the Centre Hospitalier Universitaire Vaudois (CHUV) in Lausanne, Switzerland (33,274 patients delivered between 1996 and 2011 with a 30.5% cesarean section rate). Data available in this database includes demographic, labor, and delivery information, as well as maternal and neonatal outcomes. All information was collected at the time of delivery by the obstetrician. To be eligible women had to give birth either by an elective cesarean section or a spontaneous vaginal delivery with a grade 1 vaginal tear at most. These deliveries are referred to throughout the article as the ‘index deliveries’. The exclusion criteria were as follows: women under 18 years, multiple pregnancies, instrumental deliveries. As a random selection was performed in our database, it resulted in more patients who delivered vaginally than those who delivered by c-section. The quality of this database of prospectively collected data has already been described elsewhere (cross-check congruent data in 98.2–99.8% of cases)^9^.

To project an appropriate sample size, we used studies that compared urinary incontinence rates after vaginal (23–25%) versus cesarean deliveries (10–16%)^10,11^. Based on these estimates, a sample size of 213 patients in each group would have an 80% power to detect an 11% difference with a significance level of 0.05. Based on a 35% patient response rate which we had previously observed^9^, a total of 1217 patients would be necessary to achieve the minimum sample size of 426. A similar sample size was calculated for fecal incontinence.

In order to compare the results with our previous work investigating pelvic floor function after anal sphincter tears^9^, and with other studies in the field^7,10,12^, patients who delivered 6 years before this study were randomly chosen from our hospital database. During that period, the number of vaginal deliveries exceeded the number of cesarean deliveries. For this reason, 800 uncomplicated vaginal deliveries (vaginal tears of maximal grade 1, no instrumental assistance) and 500 singleton elective cesarean deliveries were selected to reach the sample size needed (n = 1300).

Both groups received the same questionnaires by post; non-responders received reminder 2–3 months later. A total of 98 patients (7.5%) had changed address since their last consultation at our hospital. These women’s current home address was traced using phone or internet directories. Sixty-nine (5.3%) women were not located. Help for translation (n = 6) was proposed to women who were not fluent in French, either by a nurse or a midwife. All methods/experiments were carried out in accordance with relevant guidelines and regulations (Declaration of Helsinki). This study was approved by the local IRB (Ethical Commission of the Canton of Vaud, Switzerland, protocol no. 101/08). Responding to the questionnaires was considered informed consent for participation in our study.

The primary objective of this study was to report subjective pelvic floor-related symptoms (fecal, urinary and sexual) and their impact on quality of life. Patients’ current socio-demographic and physical characteristics were registered using self-reported questionnaires. Obstetrical history and mode of delivery of subsequent birth(s) (that may have occurred in other hospitals) were also collected through a self-administered questionnaire. Multiparous patients who delivered by both modes of delivery were excluded from the analysis (n = 87). Four validated questionnaires were used: the short forms of the Urogenital Distress Inventory (UDI-6) and the Incontinence Impact Questionnaire (IIQ-7)^13^; Wexner fecal incontinence scale^14^; and the Female Sexual Function Index (FSFI)^15^. The FSFI has been validated in French^16^. Concerning the UDI-6, IIQ-7 and Wexner fecal incontinence scale, no French version has yet been validated in the literature, despite being commonly used in French clinical studies. These are made available by the French urological society^17^.

The validated UDI-6 questionnaire measures the burden of incontinence symptoms, with higher scores indicating a greater degree of inconvenience or worse quality of life (Table 2)^13^. The IIQ-7 measures the impact of urinary incontinence on activities, roles, and emotional states (Table 3), with higher scores indicating a greater impact/worse quality of life. For both questionnaires, each item is categorized by the frequency of occurrence or the degree of discomfort (never, slightly, moderately and much).

Fecal incontinence was evaluated using the Wexner fecal incontinence scale^14^. The Wexner scale consists of eight items (Table 4). Each item scores between 0 and 4 related to the frequency of occurrence (0 absent, 1 less than once a month, 2 less than once a week, 3 less than once a day, 4 daily). A Wexner score of zero means absence of anal incontinence, and a score of 20 means complete incontinence. Severe fecal incontinence was defined as a Wexner score > 4.

The FSFI is a validated instrument for sexual function assessment (Table 5). This multidimensional score combines 18 questions divided into 6 subscales (desire, arousal, lubrication, orgasm, satisfaction and pain). The score ranges from 2 to 36: low scores signifying sexual impairment or little to absence of sexual activity while high scores represent high sexual activity and great satisfaction. Severe sexual dysfunction was defined as FSFI scores ≤ 25)^9^.

As we expected that most women would not report any symptoms and to avoid reporting considerably skewed distributions, we dichotomized ordinal outcomes of each questionnaire. The effects of the exposure, demographic data and risk factors were compared between both groups using the Pearson χ^2^ test (or the Fisher exact test when indicated) for categorical variables. For continuous variables, medians were compared using the Wilcoxon-Mann–Whitney test.

In addition to obstetrical exposures, we considered the following confounders: age, ethnicity, parity, weight, smoking, level of education, marital status, religion, type of health insurance.

Relative risks adjusted for these confounders were estimated by using generalized linear models (Poisson regression with robust variance estimates). Statistical analyses were performed using STATA 13.0 (Stata Corporation, College Station, USA).

## Results

Among 1231 (94.7%) women who were contacted, 604 (49%) completed the questionnaires, including 208 eCS and 309 uVD. A total of 87 patients who gave birth by another mode of delivery after the index pregnancy were excluded from the analysis. Socio-demographic and obstetrical characteristics at index pregnancy of responders and non-responders/lost patients were similar (data not shown).

Regarding the index delivery, both gestational age (39.6 versus 39.5 weeks, p = 0.57) and neonatal weight (3263 g versus 3234 g, p = 0.51) were similar between uVD and eCS groups, respectively. The indications for elective CS were breech/transverse presentations (n = 64), maternal demand (n = 58), pre-eclampsia (n = 18), low-lying placenta (n = 15), genital herpes/HIV/Hepatitis-C (n = 9), declined vaginal birth after previous cesarean section (n = 33) and other (n = 11).

Table 1 shows socio-demographic and obstetrical characteristics of both groups at the time patients returned the questionnaires. The mean time between index delivery and the submission of the questionnaires was 6.7 and 6.3 years for women having had a uVD and a eCS, respectively (p = 0.157). uVD and eCS did not exhibit any significant differences, except for marital status and religion. Data presented below are adjusted for all variables presented in Table 1. All significant items after univariate analysis remained significant after adjustment via multivariate analysis.

**Table 1:** Socio-demographic and physical characteristics of patients at the time of questionnaire completion.

Characteristics	Uncomplicated vaginal delivery (n = 309) %	Elective cesarean delivery (n = 208) %	p value
**Age [year ± SD]**	36.6 ± 5.3	37.3 ± 5.4	0.196
≥ 40 years	29.5	35.7	0.135
**Ethnicity**			
Swiss	60.9	59.7	0.784
Non-swiss	39.1	40.3	
**Parity**			
Multiparous	16.3	18.5	0.515
**Weight [kg ± SD]**	64 ± 12	66 ± 15	0.1
**Smoker**			
Yes	12.7	15.6	0.343
**University degree**			
Yes	10.1	9	0.763
**Marital status**			
Unmarried	14	23.2	0.007
Married	86	76.8	
**Religion**			
Christian	82.1	74.4	0.035
**Health insurance**			
Non-private	98.1	97.2	0.56
Private	2	2.8	

Data are shown as percentage of the total of each group, except for age and weight.

*SD* standard deviation.

The results of the UDI-6 questionnaire are presented in Table 2. When considering the total UDI-6 score, no significant difference was found in the mean score for women with uVD compared to those in the eCS group (p = 0.185). After an eCS, women were significantly less likely to be bothered by urge incontinence (adjusted Relative Risk [aRR] 0.55; 95% confidence intervals [95% CI] 0.34–0.88) and urine leakage related to physical activity (aRR 0.53; 95% CI 0.35–0.80), compared to women who had uVD. In contrast, women were more likely to complain about lower abdominal pain after eCS compared to uVD (aRR 1.58; 95% CI 1.01–2.49). Women complaining of at least one symptom (UDI-6 score ≥ 1) were significantly more frequent after uVD than eCS (76.7% versus 66.4%, p = 0.01). The results of the IIQ-7 questionnaire are presented in Table 3. Women who had an eCS reported significantly less frequent urine leakage during physical activities outside the home than women who had an uVD (aRR 0.43, 95% CI 0.20–0.92). No other significant difference was observed between both groups in the other items of the IIQ-7 questionnaire or in the IIQ-7 final score.

**Table 2:** Urinary distress inventory (UDI-6).

Moderate to great symptoms	uVD (n = 309) %	eCS (n = 208) %	p value	Adjusted RR*	95% CI
Frequent urination	38.1	30.5	0.074	0.67	0.43–1.02
Urine leakage related to urgency	32.9	22.1	0.008	0.55	0.34–0.88
Urine leakage related to physical activity	50.5	36.1	0.001	0.53	0.35–0.80
Small amounts of urine leakage (drops)	32.1	32.1	0.986	1.03	0.66–1.61
Difficulty emptying bladder	14.1	14.0	1.000	0.93	0.52–1.67
Lower abdominal or genital pain	21.0	29.7	0.025	1.58	1.01–2.49
Mean UDI-6 score	11.2	9.8	0.185		

*uVD* uncomplicated vaginal delivery, *eCS* elective cesarean section, *RR* relative risk, *CI* confidence intervals.

*Adjusted relative risks (RR) of presenting symptoms after an uncomplicated vaginal delivery compared to an elective cesarean section. Relative risks were adjusted for all socio-demographic and physical variables of Table 1.

**Table 3:** Incontinence impact questionnaire (IIQ-7).

Urine leakage during	uVD (n = 309) %	eCS (n = 208) %	p value	Adjusted RR*	95% CI
Physical activities at home	0.7	1	1	0.28	0.02–4.07
Physical activities outside home	13.4	6.5	0.017	0.43	0.20–0.92
Entertainment activities (cinema,…)	2.7	2.5	1	0.66	0.17–2.58
Travel longer than 30 min	3.4	3	1	0.57	0.17–1.97
Social activities	2.0	2.5	0.762	0.60	0.13–2.8
Feeling anxious or depressive	13.8	16	0.498	1.10	0.62–1.96
Feeling frustrated	12.1	15.6	0.27	1.18	0.65–2.15
Mean IIQ-7 score	23	22	0.902		

*uVD* uncomplicated vaginal delivery, *eCS* elective cesarean section, *RR* relative risk, *CI* confidence intervals.

*Adjusted relative risks (RR) of presenting symptoms after a uncomplicated vaginal delivery compared to an elective cesarean section. Relative risks were adjusted for all socio-demographic and physical variables of Table 1.

Regarding fecal incontinence (Table 4), all the items investigated through the Wexner questionnaire were similar between both modes of delivery, except for alteration of sexual life which was significantly more present after eCS than uVD (aRR 1.72; 95% CI 1.13–2.63). This result was confirmed through investigation of sexual life using the Female Sexual Function Index (FSFI, Table 5). Four items (no. 2, 17, 18, 19) of the FSFI were significantly worse after eCS than uVD, and 3 other questions showed similar borderline significant trends (nos. 8, 10, 16). Indeed, women who sustained eCS reported significantly more difficulties in three specific questions, relating to lubrication (nos. 8 and 10) and satisfaction (no. 16), than women in the uVD group. All items investigating pain during or following sexual intercourse were significantly worse after eCS than uVD. The score for pain was also significantly worse after eCS than uVD (p = 0.002). None of the other scores, for excitation, lubrication, orgasm or satisfaction showed significant differences. The occurrence of severe sexual dysfunction (defined as a FSFI score ≤ 25) was higher after eCS than uVD (36.2% versus 26.4%, respectively, p = 0.018).

**Table 4:** Wexner anal incontinence score.

Symptoms	uVD (n = 309) %	eCS (n = 208) %	p value	Adjusted RR*	95% CI
Incontinence for gas	39.6	38.6	0.812	0.95	0.63–1.42
Incontinence for liquid stool	10.0	11.4	0.664	1.10	0.57–2.08
Incontinence for solid stool	2.9	1.9	0.58	0.58	0.12–2.70
Alteration of lifestyle	5.5	7.1	0.45	1.04	0.43–2.54
Alteration of sexual life	1.4	4.6	0.044	1.72	1.13–2.63
Need to wear a pad	6.2	6.8	0.855	0.97	0.43–2.15
Taking constipating medicine	0.3	0.5	1	3.23	0.16–66.1
Inability to defer defecation for 15 min	9.8	13.5	0.202	1.66	0.88–3.12
Mean Wexner score	1.2	1.4	0.46		

*uVD* uncomplicated vaginal delivery, *eCS* elective cesarean section, *RR* relative risk, *CI* confidence intervals.

*Adjusted relative risks (RR) of presenting symptoms after a uncomplicated vaginal delivery compared to an elective cesarean section. Relative risks were adjusted for all socio-demographic and physical variables of Table 1.

**Table 5:** Female sexual function index (FSFI).

No	FSFI questionnaire	uVD (n = 309) %	eCS (n = 208) %	p value	Adjusted RR*	95% CI
1	Sexual desire half of the time or less	22.41	25.98	0.36	1.19	0.74–1.89
2	Low level of sexual desire	18.79	26.44	0.041	1.54	0.96–2.50
Score "DESIRE" (1.2–6)		3.84	3.7	0.178		
3	Excitation during sexual activity half of the time or less	10.3	13.46	0.324	1.27	0.68–2.33
4	Low level of excitation during sexual activity	9.97	12.98	0.318	1.06	0.56–2.04
5	Low confidence about becoming sexually excited during sexual activity	10.7	13.59	0.331	1.10	0.59–2.04
6	Satisfied with excitation during sexual activity less than half the time	9.03	12.56	0.238	1.23	0.65–2.38
Score "EXCITATION" (0–6)		4.48	4.35	0.236		
7	Lubrication during sexual activity less than half of the time	13	14.9	0.54	0.93	0.53–1.64
8	Difficulty becoming lubricated during sexual activity	18.43	24.76	0.088	1.30	0.80–2.08
9	Maintain lubrication until completion of sexual activity less than half of the time	12.75	14.49	0.573	1.03	0.57–1.85
10	Difficulty maintaining lubrication until completion of sexual activity	15.15	21.78	0.058	1.35	0.80–2.27
Score "LUBRIFICATION" (0–6)		4.83	4.7	0.274		
11	Reach orgasm less than half of the time	14.72	14.56	0.962	0.99	0.56–1.75
12	Reaching orgasm difficult	17.73	20.87	0.376	1.08	0.65–1.79
13	Moderately dissatisfied with ability to reach orgasm	15.67	18.05	0.48	1.19	0.68–2.04
Score "ORGASM" (0–6)		4.7	4.63	0.582		
14	Moderately satisfied with the amount of emotional closeness during sexual activ	16.05	18.05	0.557	1.14	0.67–1.92
15	Moderately dissatisfied about the sexual relationship	11.15	16.1	0.107	1.43	0.79–2.56
16	Moderately dissatisfied about overall sexual life	12.75	19.02	0.055	1.43	0.83–2.50
Score "SATISFACTION" (0–6)		4.39	4.26	0.283		
17	Pain during vaginal penetration about half the time or more	8.36	18.36	0.001	2.04	1.10–3.85
18	Pain following vaginal penetration more than half of the time	13.29	20.98	0.027	1.79	1.04–3.03
19	High level of pain during or following vaginal penetration	5.7	13.73	0.002	2.50	1.19–5.26
Score "PAIN" (0–6)		5.14	4.78	0.002		
Score total (2–36)		27.18	26.23	0.094		

*uvD* uncomplicated vaginal delivery, *eCS* elective cesarean section, *RR* relative risk, *CI* confidence intervals.

*Adjusted relative risks (RR) of presenting symptoms after a uncomplicated vaginal delivery compared to an elective cesarean section. Relative risks were adjusted for all socio-demographic and physical variables of Table 1.

## Discussion

We illustrated that, 6 years after the index delivery, women who underwent uVD compared to eCS more commonly experienced symptoms of urgency and stress incontinence. Although women who gave birth vaginally reported more UI, the impact of it on quality of life measured using the IIQ-7 was not different between groups (except for the specific item “Physical activities outside home”). No difference was found in the rates of fecal incontinence. The occurrence of severe sexual dysfunction was higher after eCS than uVD.

In recent years, the cesarean section rate has increased dramatically reaching 30–35% in developed countries and 50% in private practice. It is estimated that 12–15% of cesarean sections are performed upon maternal request despite potential consequences for women’s health^18^. It has been well established that an elective cesarean section compared to a vaginal delivery is associated with an increased risk of severe acute maternal morbidity (i.e. hemorrhage, complications associated with anesthetic, obstetric shock, cardiac arrest, acute renal failure, need for assisted ventilation or intubation, puerperal venous thromboembolism, infection, and hematoma)^19^. Furthermore, an increase risk of hysterectomy, abnormal placentation, uterine rupture, stillbirth, and preterm birth is found in subsequent deliveries^19^. The associate risk of urinary incontinence after vaginal delivery should therefore be balanced with the above-mentioned morbidity linked to cesarean section when counseling the patient.

In this cohort, 27.8% of eCS were performed upon maternal request. Our study was designed to evaluate the long-term symptoms of women who sustained an eCS compared to a group of women who had an uVD, in order to improve the information that is given to patients and help them make an informed choice. We acknowledge that both modes of delivery represent a “best case scenario” in comparison to emergency CS or complicated VD, which should be emphasized during patient counseling. Furthermore, we illustrated a significantly reduced risk for both urgency and stress incontinence after eCS compared to uVD. Similar associations were found by others, although some of them used urinary incontinence scoring questionnaires which were not validated^11,20–22^. Rortveit et al. found 2.3 and 1.5 fold increased risk of urinary incontinence in VD and CS patients, respectively, compared to nulliparae^21^. When comparing VD and CS together, they found a 2.4 fold increased risk only for stress incontinence and no difference for urge incontinence^21^. Gyhagen et al. also found a 1.67 fold increased risk of urinary incontinence after VD compared to CS, without differentiating between urge and stress incontinence^20^. In a previous study^9^, we showed that VD with perineal laceration conferred a 3.3 fold increased risk of pollakiuria compared to uVD, but no difference regarding incontinence.

Research on the impact of urinary incontinence on the quality of life of women after childbirth is sparse^23–26^*.* Prior studies illustrated that urgency incontinence in women following cesarean delivery more negatively impacts women’s emotional health than those who deliver vaginally, whereas stress incontinence did not significantly affect quality of life. We found no difference in the total score of the IIQ-7 between both modes of delivery. Furthermore, it has been reported in an observational study that UDI-6 scores greater than 25 provided a meaningful benchmark for care-seeking amongst women with urinary incontinence^27^. In our cohort, median UDI-6 scores for vaginal delivery and cesarean section were 11.2 and 9.8 respectively, with no significant disparity. The results concerning urinary incontinence symptoms should however be weighed against the impact on quality of life.

Our data suggest that women who sustained uVD have no increased risk of anal incontinence as measured by the Jorge and Wexner anal incontinence score and thus no alteration of their quality of life. Similar conclusions were achieved by Liebling et al.^28^ who compared instrumented vaginal delivery and emergency CS at full dilatation. However, there was a significant difference in our study for the “alteration of sexual life” item in favor of uVD compared to eCS. This difference was confirmed by the specific sexual function (FSFI) questionnaire where patients complained 2.5 times more frequently about pain during or following vaginal penetration after eCS than after uVD. Similar results were reported by McDonald et al. who followed up a cohort of 1507 nulliparae, during and 18 months after pregnancy^29^. At this later time point, there was a 2.35 fold increased risk of more intense dyspareunia after CS than after uVD. This observation could be a result of adhesions, uterine and/or abdominal scars or isthmocele, a defect in the CS uterine scar, arising after a CS. Despite many authors investigating the best surgical approach for CS in order to minimize such long-term complications, no evidence is available^30^.

Little is reported in the literature about sexual pain after CS although fear of perineal trauma and consequent sexual pain is a frequent argument of patients who request an eCS. Although pain is reported during first vaginal intercourse postnatally in the vast majority of women (85.7%), irrespective of mode of delivery, the difference between eCS and uVD seems to persist over time. However, other investigators found no difference in FSFI scores 12 months after delivery compared to the first trimester scores^31^. Several studies have been published on the prevalence of postpartum pelvic dysfunction symptoms, most notably urinary incontinence. Further long-term cohort follow-up studies are required to assess the quality of life implications of these symptoms i.e. the need to treat. Future research should also focus on the possible causes of dyspareunia after CS, investigating the role of the scar healing process.

The strength of our study was the use of validated questionnaires exploring all three pelvic floor functions and the relatively long-term results. Moreover, socio-demographic and physical characteristics of the patients were investigated and were similar in both groups. Some limitations of the present investigation must be considered: women with incontinence or sexual dysfunction may be more predisposed to participate in studies and therefore their symptoms might be overrepresented. This study lacks information on whether incontinence or sexual dysfunction were present or not before, and/or during pregnancy or started after delivery. The overall response rate of 49% may appear low in comparison to other studies^28,32–34^. However, to avoid a selection bias, we decided not to contact women by phone before sending the questionnaires, nor asked them in the immediate postpartum period to participate to this study. Similar or lower response rates (27–39%) were obtained by other authors who mailed a brief questionnaire concerning pelvic floor symptoms to an unselected group of women after vaginal birth^7,8,22^. Additionally, no data were available regarding whether patients had pelvic surgery, pelvic floor muscle training, behavioral therapies or other types of treatment, all of which are factors that may influence the symptoms reported by the patient. In line with other large cohort studies on pelvic floor dysfunction, we used self-report questionnaires, and patients were not clinically evaluated to confirm the findings. We believe that self-report questionnaires highlight the symptoms which are most consequential for the patient. We must also be mindful that patient-reported symptoms may not directly correlate with a clinician’s diagnosis, and so our results should interpreted in this light. Finally, we wished to determine the effect of the actual mode of delivery rather than the effect of the approach to delivery. Our results were thus not analyzed according to intention to treat.

## Conclusion

Our study showed that women after uVD were more likely to report urinary incontinence than eCS. In contrast, women after eCS more frequently reported sexual dysfunction symptoms in particular with more painful intercourse than those who underwent uVD. This study highlights pros and cons for each type of delivery, thus providing clinicians with a decision tool to better inform pregnant women about delivery’s long-term consequences, especially in instances of CS on maternal request.
